# A FTIR Imaging Characterization of Fibroblasts Stimulated by Various Breast Cancer Cell Lines

**DOI:** 10.1371/journal.pone.0111137

**Published:** 2014-11-12

**Authors:** Saroj Kumar, Thankaraj Salammal Shabi, Erik Goormaghtigh

**Affiliations:** 1 Laboratory for the Structure and Function of Biological Membranes, Center for Structural Biology and Bioinformatics, Université Libre de Bruxelles, Brussels, Belgium; 2 Organic Semiconductor Lab, Department of Polymer Science and Engineering, Zhejiang University, P. R. China; Wayne State University School of Medicine, United States of America

## Abstract

It is well known that the microenvironment plays a major role in breast cancer progression. Yet, the mechanism explaining the transition from normal fibroblasts to cancer-stimulated fibroblasts remains to be elucidated. Here we report a FTIR imaging study of the effects of three different breast cancer cell lines on normal fibroblasts in culture. Fibroblast activation process was monitored by FTIR imaging and spectra compared by multivariate statistical analyses. Principal component analysis evidenced that the fibroblasts stimulated by these cancer cell lines grouped together and remained distinctly separated from normal fibroblasts indicating a modified different chemical composition in the cancer-stimulated fibroblasts. Similar changes in fibroblasts were induced by the various breast cancer cell lines belonging to different sub-types. Most significant changes were observed in the region of 2950 and 1230 cm^−1^, possibly related to changes in lipids and in the 1230 cm^−1^ area assigned to phosphate vibrations (nucleotides). Interestingly, the cancer-cell induced changes in the fibroblasts also occurred when there was no possible direct contact between the two cell lines in the co-culture. When contact was possible, the spectral changes were similar, suggesting that soluble factors but not direct cell-cell interactions were responsible for fibroblast activation. Overall, the results indicate that IR imaging could be used in the future for analyzing the microenvironment of breast tumors.

## Introduction

In western woman, breast cancer has the highest rate of incidence and is the second major cause of mortality [1], [2]. Most of the deaths of these patients are due metastasis where cancer cells spread outside the ducts into the extracellular matrix, then later migrate to blood vessels and the lymphatic system. Early diagnosis and application of adjuvant therapy is the key to prevent metastasis. There are several adjuvant therapies such as radiotherapy, chemotherapy, antibody therapy that target the cancer cells and preserve as much as possible the stromal cells present in the surrounding environment [1]. The stroma includes extracellular and cellular tissue network which interact with the cancer cells. It undergoes significant modifications in the presence of the cancer and becomes the so-called reactive stroma [3]–[10]. In early growth of tumors, cancer cells are confined within the boundary of the basement membrane which acts as a gatekeeper [1], [10], [11] and stroma is considered to be a passive responder to invasiveness [1], [9], [12], [13]. The mechanism involved in the progression towards metastasis remains elusive but it is demonstrated that profound changes occur in breast stromal fibroblasts present in histologically normal surgical margins [14]. In particular, these fibroblasts have been shown to secrete pro-carcinogenic cytokines. Yet, the interactions by which the cancer cells influence the normal stroma, which then becomes a “reactive stroma”, remain poorly described. The role of reactive stroma in initiation of invasiveness is described but also poorly understood. In particular, differences in chemical signatures of stromal tissue close to the tumor and far from the tumor (normal tissue) have been evidenced and could provide clues for understanding tumorgenesis [1], [2], [15].

Stimulated fibroblasts, also known as myofibroblasts, are associated with cancer cells and known as cancer associated fibroblasts (CAFs). Earlier studies [9], [10], [16]–[19], show that, by comparison with the normal fibroblasts, CAFs initiate and enhance invasiveness by secretion of several growth factors (hepatocyte growth factor, transforming growth factor etc.) and extracellular matrix (ECM) proteins (e.g. metalloproteinase). The specific role of CAFs in tumorogenicity in mouse models [20], [21] and in in-vitro culture [10], [17] has been described. An obvious change observed in the CAFs is the high expression of α- smooth muscle-actin stress fibers (α-SMA) [9], [22] but a later study [17] showed that α-SMA does not play any significant role in tumor growth. Understanding the metastasis process could be significantly improved if cancer cell interactions with the microenvironment were better known. Recent studies suggest that stroma could be an effective therapeutic target for epithelial tumor [9], [10], [17], suggesting that more emphasis should be given to the role of CAFs [9].

Genomic level characterization is a promising tool to provide individual treatment but remains expensive and is limited by the difficulty to achieve this characterization at cell level as tumors display extended heterogeneity [22], [23]. It is therefore necessary to work towards the development of methods to characterize the stroma during tumor progression at cell level [15] and further stress should be given to fibroblast [4], [22], [24]. Infrared spectroscopy monitors the global chemical composition of the sample. A spectrum of cells is a superimposition of spectra of all cell constituents [25], [26]. Furthermore, the IR spectra account not only for the chemical nature of cell molecules but also for their conformations and are, in particular, very sensitive to protein secondary structure [27]–[29]. In particular, modifications of collagen structure have been demonstrated by FTIR spectroscopy [30]–[32]. FTIR imaging has been used extensively to study biochemical changes within cells, for instance during the cell cycle [33]–[36], upon addition of anticancer drugs [37]–[41], as well as on different cell lines [42], [43]. Previous FTIR studies on fibroblasts [22], [26], [44] have evidenced FTIR spectral modifications of their FTIR spectra when fibroblasts were placed in the presence of either TGFβ1 or MCF-7 breast cancer cell line. These changes were correlated with the production of α-smooth muscle actin (α-SMA) by the fibroblasts in response to soluble proteins. Here we used the label free FTIR imaging technique to study the effects of different breast cancer cell lines on normal fibroblasts in culture.

## Material and Methods

### Cell lines and growth conditions

The fibroblast cell line (CCD-1126Sk) established from a normal human breast was obtained from the American Type Culture Collection (ATCC). Breast cancer cell lines MCF-7, MDA-MB-321, SK-BR-3 were also obtained from ATCC. The characteristics of these cell lines are presented in table 1. Iscove's Modified Dulbecco's Media (IMDM), fetal calf serum (FCS), trypsin, penicillin and streptomycin were purchased from Lonza (Belgium).

**Table 1:** Characteristics of breast cell lines used: estrogen receptor (ER), progesterone receptor (PgR), human epidermal growth factor receptor 2 (ERBB2) amplification status.

Cell line	ER	PgR	ERBB2 amp.	Tumorigenicity	Tumor classification
MCF-7	+	+		Yes	Luminal
MDA-MB-231	_	_		Yes	Basal B
SK-BR-3	**_**	**_**	+	Yes	Luminal

Tumorigenicity and tumor classification is based on published data [58]–[60].

All cell lines were grown in the conditioned media (CM) which contains: IMDM supplemented with 10% FCS, and 1% penicillin-streptomycin in a humidified incubator supplied with 5% CO_2_ at 37°C [45]. Different cell lines were grown in T75 or T25 flasks and the medium was changed every 2–3 times/week, when cells were reaching sub-confluence. Detachment of the cells was achieved by means of a five-minute treatment with 1 ml trypsin (0.5 g/l)/EDTA (0.2 g/l) buffer (Lonza, Verviers, Belgium). The reaction was stopped by adding 1 ml of culture medium.

**Figure 1:**
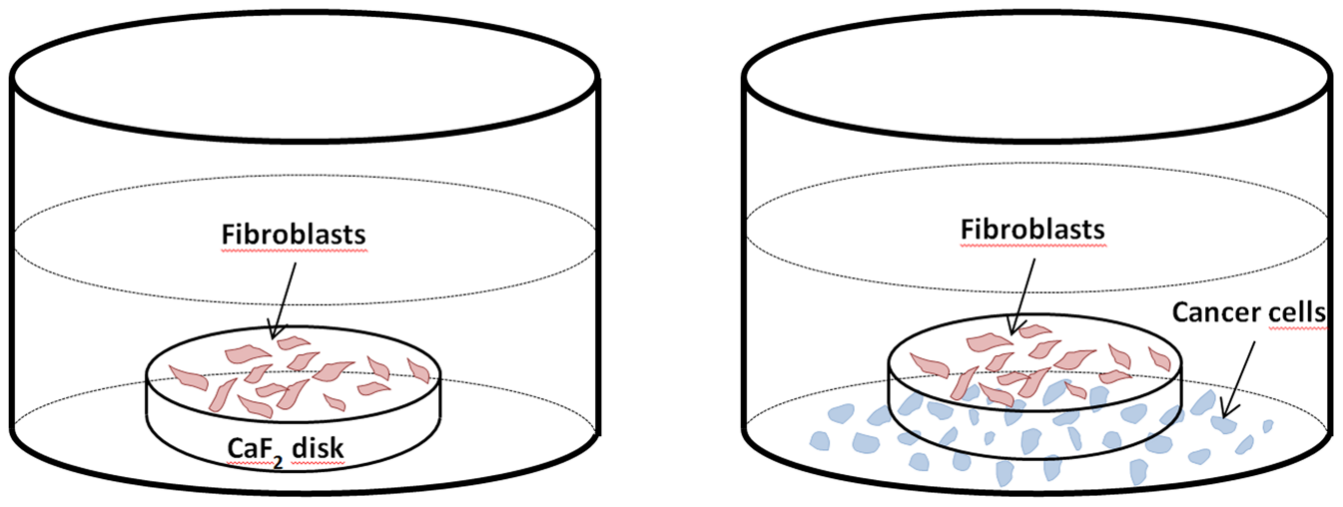
Schematic presentation of 2 wells from a 12-well culture plate. On the left-hand side well, fibroblasts were grown alone on a CaF_2_ disk. On the right-hand side well, the CaF_2_ disk bearing the fibroblasts has been transferred into a well where cancer cells are growing. No direct contact between the two cell types is possible but soluble factors can diffuse through the solution.

We designed a method for co-culture the fibroblasts with different breast cancer cell lines (see Fig. 1) with either non-contact between fibroblasts and cancer cells or with contact. For non-contact cultures, we grew the fibroblasts on a CaF_2_ window (transparent to IR) and cancer cell lines on the bottom of a 12 well plate culture box. Both cell types were first grown for 6 hours in different wells to let the cells settle on the surface. In a second step, the CaF_2_ window with the fibroblasts attached to it were transferred into the wells where cancer cells were growing. The length of the fibroblasts was measured by the Leica Microsystems microscope imaging software. Prior to FTIR measurements the cells were briefly washed on CaF_2_ window by distilled water. Drying was obtained either by using a gentle dry air flow or by letting the cell dry with the CaF_2_ windows put vertically. Visual inspection under the microscope indicated that the cells were intact in both cases. Furthermore, the IR spectra collected in the space between the cells did not contain organic materials which could indicate cell leakage. Even though it has been shown that fixation by drying or by formaldehyde followed by inclusion into paraffin largely preserves spectral differences [45], drying was preferred here as it does not require cell detachment from the CaF_2_ substrate. In turn, cell shape was partially preserved. For the few experiments where contact between fibroblasts and cancer cells was allowed, the two cell types were let to settle together on the CaF_2_ window. In all cases, the cells dried on CaF_2_ window were examined immediately. Storage at room temperature for 3-5 days neither changed their visual aspect nor their infrared spectra.

### IR measurement

The FTIR data were acquired using a Hyperion 3000 FTIR imaging system (Bruker Optics, Ettlingen, Germany) equipped with a liquid nitrogen cooled 64*64 MCT (Mercury-Cadmium-Telluride) 2560×2560 µm^2^ FPA (Focal Plane Array) detector, with a 15× magnification. The spectral resolution was set to 8 cm^−1^. The data were acquired in transmission mode from sample regions of 170*170 µm^2^ for each unit image. Transmission mode is less prone to spectral distortion than transflection mode [46], [47]. Each individual element of the array detector sampled an area of 2.7*2.7 µm^2^. One FTIR unit image resulted in 4,096 spectra, each one being the average of 256 scans recorded in about 5 minutes. The experiments were performed at room temperature using the Bruker spectrometer software OPUS. Each single beam spectrum was ratioed against a background spectrum obtained in the absence of sample and converted to absorbance by OPUS. Mapping larger areas (typically 3×3 or 3×4 unit images) were usually achieved. All spectral processing and classifications were carried out using the program Kinetics written by our group and running under Matlab (Mathworks Inc, Natick, Ma, USA). All the spectra were preprocessed as described previously [28], [29]. The water vapor contribution was subtracted as described previously [48], [49] with 1956–1935 cm^−1^ as reference peak. Spectra were normalized for equal area between 1725 and 1481 cm^−1^. An 11-point baseline passing by 3620 2995 2800 2395 2247 1765 1724 1480 1355 1200 cm^−1^ was subtracted [50]. A systematic study was conducted in 2006 on the impact of baseline correction and derivatization on the discrimination power and it was found that, as long as discrimination is the issue, all approaches yielded similar results [51].

### Statistical Analyses

Principal Component Analysis (PCA) is an unsupervised multivariate method, enabling a variable reduction by building linear combinations of spectral absorbances, called Principal Component (PC), that co-vary in the selected series of spectra. The first principal component explains most of the data variance. The second principal component, uncorrelated to the first one, accounts for most of the residual variance and so on. The projection (score) of each spectrum on the different PCs indicates the importance of the contribution of that PC to the spectrum. Student's t-test was computed at every wavenumber with a significance level of α = 0.001 and a Bonferroni correction for multiple comparisons. Each marked red star indicates a statistically significant difference between the means.

## Results and Discussion

Fibroblasts were grown for 48 hours on CaF_2_ window in the presence or absence of a cancer cell line but with no direct contact between the cell types as explained in Materials and Methods and Fig 1B. We observed that the shape and size of the fibroblasts were significantly modified by the presence of the cancer cell lines. Figure S1 presents pictures illustrating live fibroblasts in the growth medium in the presence and absence of MCF-7 cells respectively as well as pictures illustrating fibroblasts in the presence and absence of MDA-MB-231 cells respectively. Statistics on cell length is also shown (mean plus/minus standard deviation) in Figure S1. The MCF-7 stimulated fibroblasts got a spindle shape and the overall size of the cancer-stimulated fibroblasts was typically increased by ca 30–50%. In particular, the length of some of these fibroblasts increased by 2–3 folds, in agreement with the appearance of stress fibers described elsewhere [26]. In the presence of MDA-MB 321, the size of cancer-stimulated fibroblasts increased by only 10–15% but the spindle shape was similar to the one observed in the presence of MCF-7. We did not observe significant changes in the size when normal fibroblasts were co-cultured with SKBR-3 cancer cells but the shape became more spindle-like. These observations indicate that fibroblasts are stimulated by the different cell lines, even though possibly to different extents, through soluble factors present in the medium. A FTIR spectroscopy study of these fibroblasts stimulated by different breast carcinoma cell lines could reveal the presence of different phenotypes or of different extent of cell content modifications.

### IR analysis of fibroblasts stimulated with three different cancer cell lines in the absence of direct contact between the cell lines

**Figure 2:**
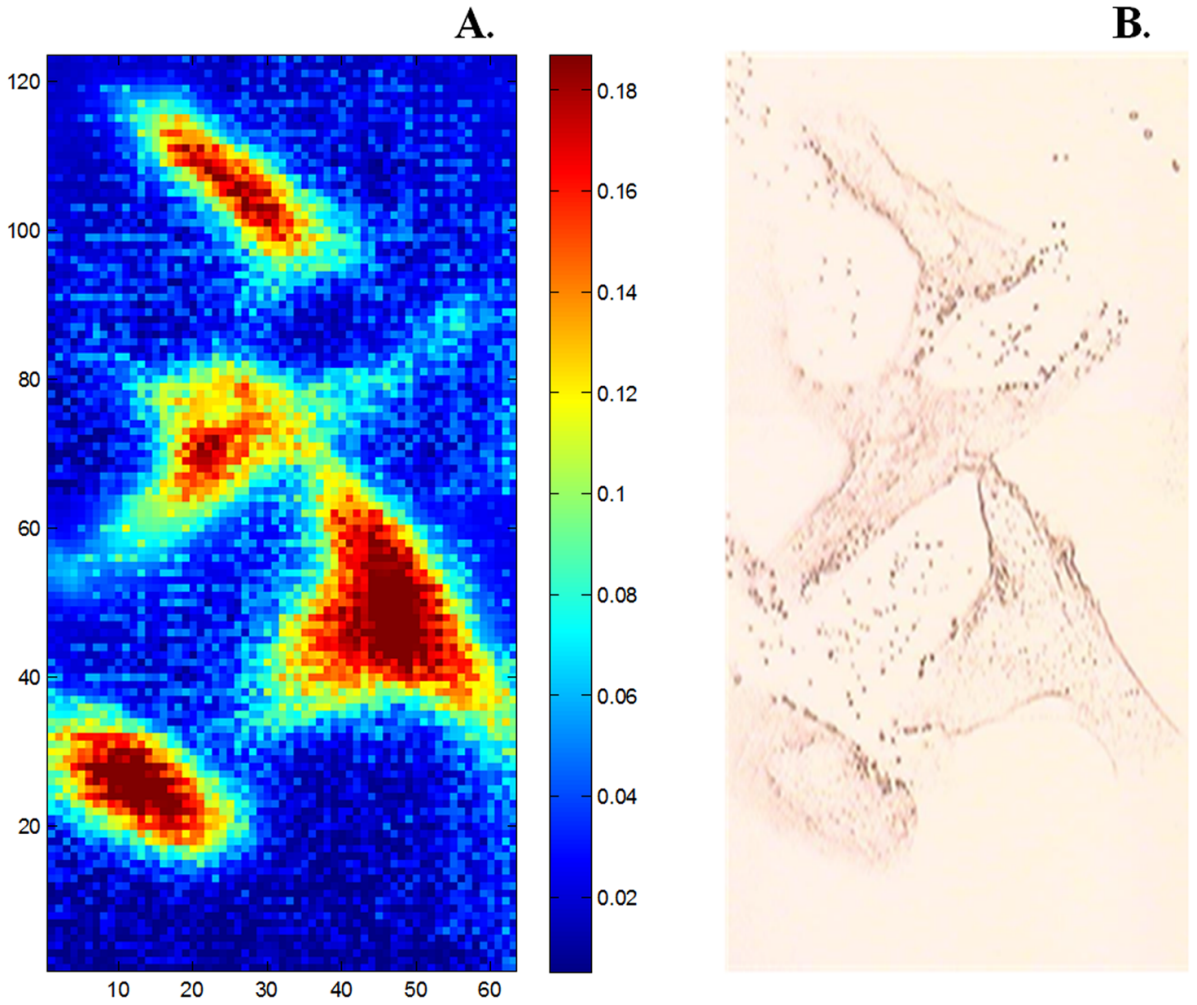
Example of IR and bright field fibroblast iamges (A) Typical IR image of fibroblasts generated by using the infrared absorbance of 1654 cm^−1^. Pixel size is 2.7×2.7 µm^2^. (B) Bright field microscopy image of the same sample.

**Figure 3:**
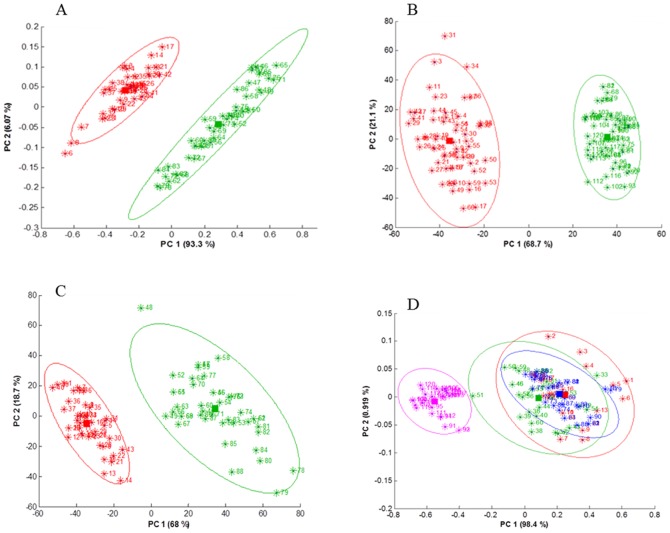
Principle component analyses of fibroblasts spectra. A, B and C: PCA score plot of spectra from fibroblasts alone (red) and fibroblasts stimulated (green) by the cancer cells MCF-7, MDA-MB-231 and SKBR-3 respectively. The scores on the first two principal components are represented. (D) PCA score plot of all spectra present in A, B and C. Fibroblasts grown alone (pink) and in the presence of the cell lines MCF-7 (red), MDA-MB-231 (blue) and SKBR-3 (green) have been submitted to the same PCA. Ellipses are drawn at 95% confidence interval. The region used for PCA was 1800–1485 cm^−1^.

A typical raw IR image representing the absorbance at 1654 cm^−1^ is reported in Fig. 2. Fig. 2A represents the IR absorbance at 1654 cm^−1^ for fibroblasts grown with no direct contact with MCF-7 cells. A bright field microscopy corresponding image is reported in Fig. 2B. Multivariate statistical analysis, PCA (Principal component analysis) was used to evidence changes in fibroblast spectra resulting from the presence of the various breast cancer cell lines. Unless otherwise mentioned, all experiments were carried out with no direct contact between fibroblasts and cancer cells (Fig 1, right panel). Fig. 3A shows a score plot where each point represents a spectrum projected in the PC1–PC2 space. For the clarity of the figure, only 45 spectra (one spectrum corresponds to a 2.7×2.7 µm pixel of the image even though it must be stressed that this size is much smaller than the diffraction limited resolution [52]) were randomly selected from fibroblasts grown alone (red) or stimulated (green) by MCF-7 (Fig 3A), MDA-MB-231(Fig 3B) and SKBR-3 (Fig 3C) cell lines. For all cell line tested, there is an obvious grouping of unstimulated and stimulated fibroblasts. The first two PC accounted for the largest part of the total variance. When analyzed together (Fig 3D) we observed that IR spectra from fibroblasts stimulated by the different cancer cell lines (MCF-7, MDA-MB-321, SKBR-3) group together but are distinctly separated from unstimulated fibroblasts. The first two PC (representing 98.4 and 0.9% of the total variance respectively) accounted together for over 99% of original variability. The grouping of all stimulated fibroblast IR spectra is indicative of similar changes induced by all breast cancer cell lines. The fact that they cluster together when projected on the first two PCs accounting together for 99.3% of the total variance indicates that the chemistry of the cells is very similar, in spite of significant changes of their size.

**Figure 4:**
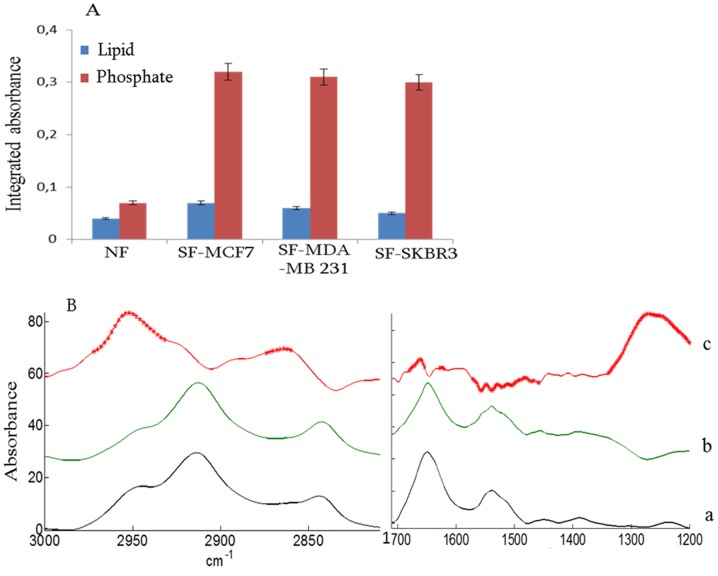
Comparison of lipid and phosphate changes observed in fibroblast grown alone (NF) or stimulated (SF) by cancer cell lines MCF-7, MDA-MB-231 and SKBR-3 (A.). Error bars indicate plus or minus one standard deviation. (B) Mean spectra of fibroblasts grown alone (a) and stimulated by MCF-7 cells (b) and difference between the means (stimulated-normal) (c). A Student t-test was computed at every wavenumber with a significance level of α = 0.01% and a Bonferroni correction for multiple comparisons. Each red star indicates a statistically significant difference between the means.

Typical spectral markers illustrating the amplitude of the changes induced by the various cancer cell lines are reported in Figure 4. Fig 4B reports the mean of the fibroblast spectra when grown in the presence (a, black) or in the absence (b, green) of MCF-7 cells. Curve c corresponds to the difference curve a - curve b, rescaled for a better visualization of the differences. A Student t-test was performed with the full dataset at every wavenumber. The stars reported on the difference spectrum indicate wavenumbers where the Student t-test rejects the equality of the means with α = 0.1% and a Bonferroni correction for multiple comparisons. The t-test reveals significant differences in the band at 2950 and 1230 cm^−1^ as well as some features including amide I and amide II ranges. The major changes observed at 2950 and 1230 cm^−1^ could originate from ν_as_(CH_3_) and P-O stretching vibrations tentatively assigned to lipids and nucleic acids respectively. The ν(CH_3_) (ca 2950 cm^−1^) to ν(CH_2_) (ca 2920 cm^−1^) ratio is an index of lipid acyl chain length [53], [54]. Higher absorption at these wavenumbers for stimulated fibroblasts may be indicative of accelerated cell growth and division. These observations are consistent with an altered chemical composition of fibroblasts upon stimulation by cancer cell line [10], [17], [22]. These changes are better visualized when the two bands discussed above are integrated (Fig. 4A). The band area assigned tentatively to lipid and phosphate are almost 6–7 times larger for stimulated fibroblasts than for fibroblasts alone.

### Comparison of fibroblasts grown in direct and indirect contact with cancer cell lines

**Figure 5:**
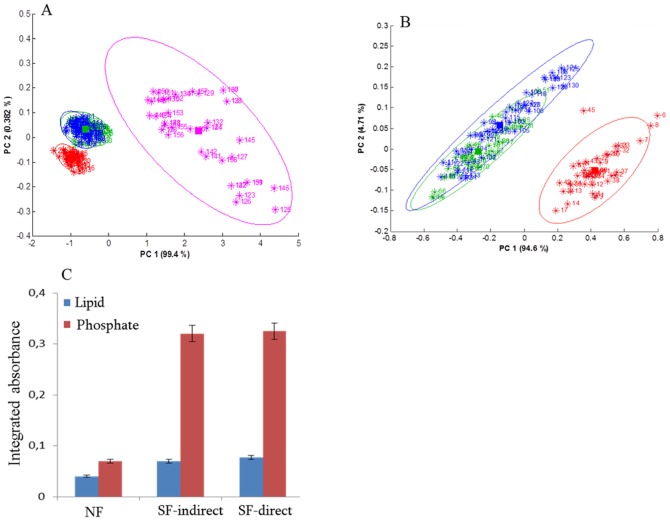
Evaluation of direct and no-contact interactions between fibroblasts and MCF-7 breast cancer cells (A) PCA score plot of spectra from normal (red), cancer stimulated with no direct contact (green) and with direct contact (blue) fibroblasts and of the MCF-7 (magenta). The spectral region used for PCA was 1800–1485 cm^−1^. (B) PCA score plot of spectra from normal (red), cancer stimulated (MCF-7) in indirect contact (green) and direct contact (blue) with fibroblasts. Ellipses are drawn at 95% confidence interval. (C) Plot represents the lipid and phosphate changes observed in normal fibroblast (NF), cancer stimulated fibroblasts not in contact (SF indirect) or with direct contact (SF direct). Error bars indicate plus or minus one standard deviation.

Figure 5 presents a PCA score plot of fibroblast spectra acquired in different conditions. For the sake of the clarity, 40 spectra were randomly selected from normal fibroblasts (red), 40 spectra from stimulated fibroblasts (no contact with cancer cell lines) (green), 40 spectra from stimulated fibroblasts (blue) in the direct contact with the MCF7 cancer cell lines and 40 spectra of the MCF-7 cells themselves (magenta). In figure 5A, the first two PC accounted for 99.4 and 0.38% of the total variance respectively. We observed a first grouping of fibroblasts on the one hand and MCF-7 cancer cell lines on the other hand (Fig 5A). If the cancer cells are removed from the analysis (Fig 5B), fibroblasts grown alone are clearly distinct from one other group containing directly and indirectly stimulated fibroblasts but there is no significant difference related to the mode (direct or indirect) of contact between fibroblasts and the MCF-7 cells. These changes are better visualized when the bands assigned to lipids and phosphate are integrated (Fig. 5C). We observed similar changes in lipid and phosphate amount in the cancer-stimulated fibroblasts (either directly or indirectly). The same behavior was observed for the other cell lines.

The biological significance of these results is difficult to assess. As spectra were all scaled on a protein band, the increase in lipid and phosphate band intensity is relative to a same protein amount. Modification of lipid synthesis has been widely demonstrated in cancer cells [1], [55] but not yet for fibroblasts. It could be part of the transformation undergone by the fibroblasts in the course of the stimulation. The phosphate band at 1230 cm^−1^ could arise from the phosphate backbone of nucleic acid. The higher phosphate absorption in stimulated fibroblast potentially indicates a less compact nucleic acid materials related to increased nucleic acid synthesis activity. It has been demonstrated that the very compact DNA has an overall decreased absorbance, not because of the presence of less DNA molecules but mainly because of the local high concentration and loss of Beer law linearity [34], [56].

## Concluding Remarks

FTIR spectroscopy imaging is providing spatially resolved chemical information which could add a significant value to current cancer diagnostic assessment. We observed here that fibroblasts grown alone experience very significant modifications when placed in the presence of breast cancer cell lines. In addition to changes in their shape and size, IR imaging and statistical analyses revealed that three different cancer cell lines which have different characteristic in terms of cancer classification (ER, PgR, ERBB2 amplification, summarized in table 1) induce similar modifications of the fibroblasts IR spectra. In a previous work [15] we showed in breast cancer tissue sections that normal stroma had a IR spectrum different from the stroma close to the cancer. In the frame of the investigation of tumor margins [57], analysis of the extracellular matrix is becoming important and an exponential decay of some spectral characteristics was observed as a function of the distance to the tumor [15]. The infrared-based chemical imaging approach to analyze and discriminate the normal and cancer stimulated fibroblasts could be used in the future for improving diagnostics of breast cancer in pre-invasive stages, before changes become observable by conventional method. Understanding tumor-induced microenvironment changes could lead to novel therapies. It must be stressed that infrared spectroscopy cannot compete with gene expression profiling as far as discovery of molecular mechanism is concerned. It lacks the specificity of molecular identification. The unique advantage of FTIR imaging is that it is very sensitive and data can be acquired with a relatively high spatial resolution. Imaging allows thousands or millions of cells to be analyzed very rapidly with no reagent.

## Supporting Information

Figure S1 Bright light microscopic image of normal fibroblast (A and D) and fibroblasts co-culture respectively with MCF7 (B) and MDA-MB-231 (E) cancer cell line grown on CaF2 window in conditioned media (IMDM). In co-cultures there was no direct contact with the cancer cells. Arrows in B indicate markedly elongated cells due to effects of MCF7 on fibroblasts. C and F report a statistical analysis of the length of the fibroblasts in the absence and in the presence of cancer cells respectively. Error bars indicate plus or minus one standard deviation.
(TIF)
