# Supplementary figures and images for: A FTIR Imaging Characterization of Fibroblasts Stimulated by Various Breast Cancer Cell Lines
Source: PLoS One. 2014 Nov 12;9(11):e111137. doi: 10.1371/journal.pone.0111137 (PMC4229076; doi:10.1371/journal.pone.0111137)

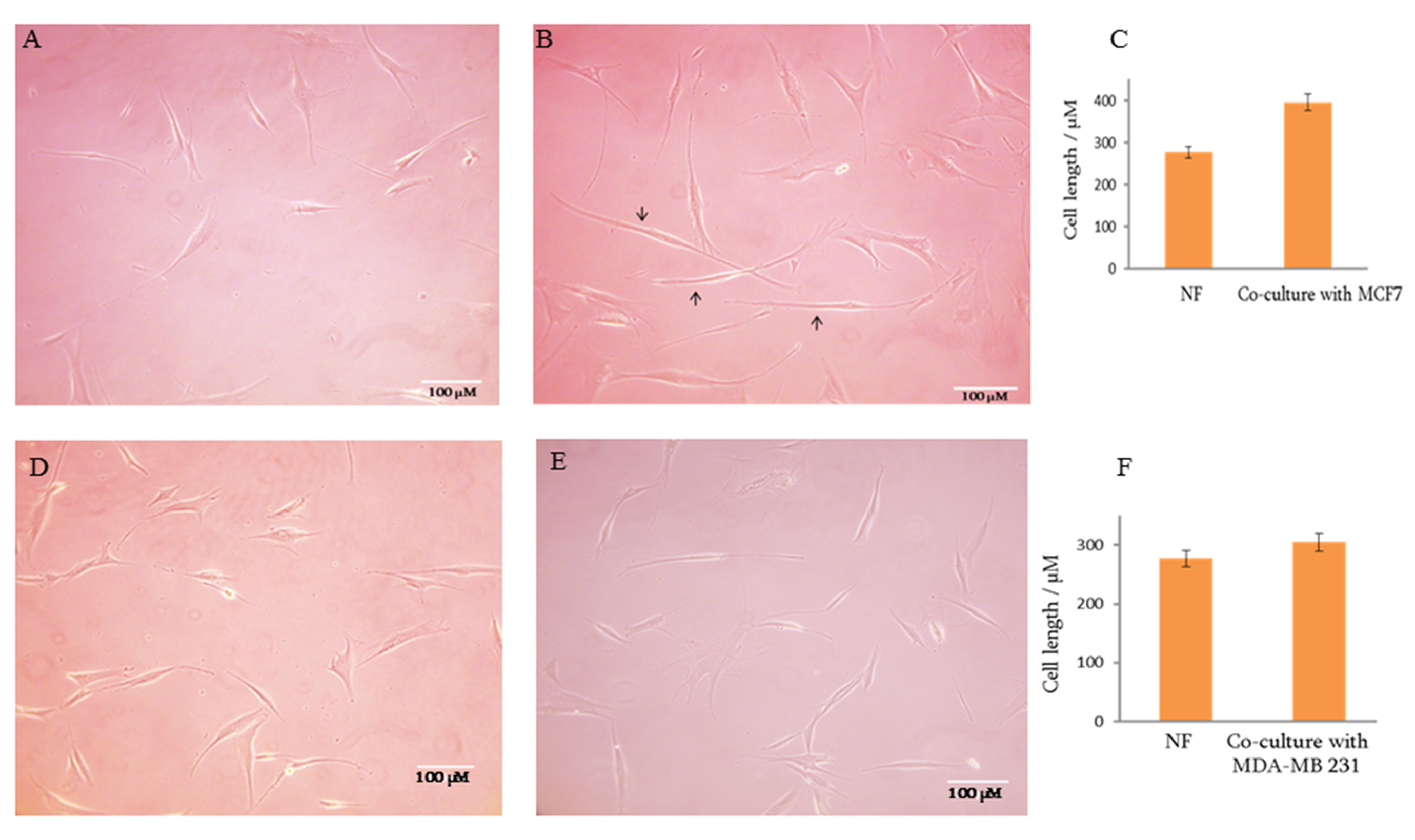

Supplement: Figure S1 — Bright light microscopic image of normal fibroblast (A and D) and fibroblasts co-culture respectively with MCF7 (B) and MDA-MB-231 (E) cancer cell line grown on CaF2 window in conditioned media (IMDM). In co-cultures there was no direct contact with the cancer cells. Arrows in B indicate markedly elongated cells due to effects of MCF7 on fibroblasts. C and F report a statistical analysis of the length of the fibroblasts in the absence and in the presence of cancer cells respectively. Error bars indicate plus or minus one standard deviation. (TIF) [file pone.0111137.s001.tif]
